# B-phosphatidylethanol testing to identify hazardous alcohol use in primary health care—a game changer and a challenge for general practitioners: a qualitative study

**DOI:** 10.1080/02813432.2025.2456949

**Published:** 2025-01-29

**Authors:** Åsa Steensland, Anna Segernäs, Mårten Larsson, Andrea Johansson Capusan, Lisa Kastbom

**Affiliations:** a Center for Social and Affective Neuroscience, Department of Biomedical and Clinical Sciences, Linköping University, Linköping, Sweden; b Primary Health Care Centre Ekholmen, Linköping and Department of Health, Medicine and Caring Sciences, Linköping University, Linköping, Sweden; c Department of Psychiatry in Norrköping and Department of Biomedical and Clinical Sciences, Linköping University, Linköping, Sweden; d Department of Psychiatry in Linköping and Department of Biomedical and Clinical Sciences, Linköping University, Linköping, Sweden

**Keywords:** Addiction medicine, alcohol dependence, alcohol misuse, alcohol use disorder, phosphatidylethanol, primary health care, qualitative research

## Abstract

**Background and aims:**

Alcohol use disorder (AUD) and hazardous alcohol use are common but underdiagnosed in primary health care (PHC). This study aimed to explore general practitioners’ (GPs’) experiences and perceptions of using B-Phosphatidylethanol (PEth), a specific quantitative biomarker for alcohol use, in their clinical work with patient consultations and treatment follow-up in Swedish PHC.

**Design, participants, and setting:**

Individual interviews were conducted with GPs and resident GPs (n 20) in Swedish PHC and analysed using qualitative content analysis.

**Findings:**

The overarching theme *PEth testing in primary health care—a game changer and a challenge* illustrated that PEth testing has improved the prerequisites for the GP-patient interaction while making it more complex. Four categories underpinned this theme: *Comprehending the context*, describing the challenges in the GP-patient interaction when hazardous alcohol use or AUD was suspected; *Getting the pieces in place*, illustrating the struggle of integrating PEth testing into clinical practice and how it diminished the role of alcohol history taking; *The challenges and facilitators of the conversation*, comprising both the difficulties in informing about PEth testing and the positive impact on the interaction, and *Considerations based on the PEth test results*, emphasising the consequences of elevated PEth test results and their influence on physicians’ motivation to using PEth.

**Conclusions:**

PEth is an important tool in the identification of hazardous alcohol use. Emerging ethical dilemmas regarding patient information on PEth testing and management of medical and medico-legal obligations when test results indicate high alcohol use need to be addressed in future guidelines for clinical management of PEth.

## Introduction

Alcohol use is a major cause of morbidity and mortality [1–3], contributing to around four percent of the global burden of disease [4]. Both detection of hazardous alcohol use as well as diagnosis and first-line treatment of mild to moderate alcohol use disorder (AUD) and of related diseases, are provided in primary health care (PHC), both internationally [5], and according to the Swedish National Board of Health and Welfare [6]. Detection of hazardous alcohol use and AUD in PHC is low. In Sweden, when AUD is diagnosed in PHC, patients are often referred to specialist care, and a recent report indicates that only 10% of patients with AUD are offered medical treatment [7].

While hazardous alcohol use denotes a quantity or frequency of use associated with negative health outcomes, AUD is a psychiatric disorder characterised by impaired control over drinking together with other physical and behavioural symptoms [8]. Evidence suggests that structured screening could increase detection of these conditions in PHC [9], while simultaneously decreasing stigma. However, questions about alcohol habits are considered to be sensitive and potentially stigmatising both by patients and general practitioners (GPs) [10]. After decades of recommendations, the use of screening questionnaires such as the Alcohol Use Disorders Identification Test (AUDIT) [11] remains low. The limited use of the AUDIT has been attributed to time constraints, lack of training and a perceived lack of treatment options [12–14, 5].

Previous research indicates that patients in PHC may be open to the use of biomarkers [15]. While heavy drinkers may be embarrassed by questions regarding alcohol use [16], there is no evidence that they would object to laboratory screening [15]. Several tests, with different strengths and limitations, have been used as indirect markers of alcohol use. A highly sensitive, theoretically 100% specific biomarker for alcohol intake, B-Phosphatidylethanol (PEth) [17,18], first introduced in Sweden in 2006 [17], could help GPs detect hazardous levels of alcohol use [17,19,20]. Since it is a quantitative marker, it could also be used to monitor treatment effect. PEth blood concentration correlates to the amount of alcohol used, where <0.05 µmol/L is interpreted as low or no alcohol intake, 0.05–0.3 µmol/L as moderate to high use and >0.3 µmol/L as high and regular alcohol use [21]. In this paper we refer an elevated test value to PEth >0.3 µmol/L, which is the unit and cut-off used in Sweden. A recent study suggested lower cut-off [22], but there is no consensus yet regarding these limits [23].

Although PEth has been widely available since 2014 [24], its use in PHC is still limited and varies significantly between PHC centres (PHCCs), based on regional laboratory statistics provided by the local health care provider (unpublished data). The reasons for this low and uneven use are unclear. GPs’ perspectives on the use of this specific alcohol biomarker can reveal obstacles and opportunities in the broader implementation of clinical tools. They can inform both training programmes on AUD for GPs and policies in PHC with the goal of improving healthcare for patients suffering from AUD.

Studies of physicians’ experiences and perceptions of PEth testing are scarce. One previous study focused on hypertension treatment and GPs’ experiences of the utility of PEth [25]. In the present study, we aimed to expand the scope to GPs’ experiences and perceptions of using PEth testing in their clinical work with patient consultations and treatment follow-up in Swedish PHC.

## Methods

### Participants and interviews

We first obtained data on PEth usage in all PHCCs from the central laboratory service in the region. We then included three PHCCs with high (7 participants), two with moderate (4 participants) and five with low PEth usage (9 participants) (Table 1), a classification made after assessment by the research group. All GPs and resident GPs working at these purposively selected PHCCs in both urban (n 6) and rural (n 4) areas with varying socio-economic conditions in southeastern Sweden were invited to participate after permission from the heads of the PHCCs.

**Table 1.** PEth usage at the PHCCs from where the participants were recruited.

PHCC	PEth/1000 inhabitants/1 year	PEth use at PHCC
1	54	High
2	13	Low
3	9,6	Low
4	63.5	High
5	14.9	Low
6	6.9	Low
7	40.3	High
8	8.2	Low
9	25.7	Moderate
10	23.6	Moderate

Since all the invited potential participants fulfilled the inclusion criteria, it was possible to include everyone who was positive to participate. We subsequently contacted selected individuals working at the PHCC units as a second invitation, to broaden the variety regarding age, gender and work experience on different types of PHCCs. All the invited potential participants agreed to participate. However, one of them declined participation before conducting the interview.

Participants (*n* 20; 6 men and 14 women, aged 31–66) were recruited. The inclusion criteria were working as a GP or a resident GP (hereafter GP) at one of the selected PHCCs, experience of PEth use in PHC and consent to digital recording during interviews. The participants were not given information about PEth use (high, moderate or low) at their PHCCs. The researchers who conducted the interviews did not have any statistical information regarding each participant’s individual PEth use in their clinical work. Written informed consent was obtained before the interviews.

The research group developed an interview guide (^Appendix^) with open-ended questions about the participants’ experiences and perceptions of PEth testing in PHC. The interview guide was tested in two pilot interviews, which were conducted by two physicians: one GP, PhD student (ÅS; female) and one resident in psychiatry (ML; male). This was followed by a consensus discussion in the research group. No subsequent corrections of the interview guide were made. Interviews continued until data redundancy [26] was reached, i.e. conducting additional interviews was not considered to add new views and perceptions, which was discussed between LK (female, MD, GP, PhD), ÅS and ML The two pilot interviews were included in the study since the quality was considered high and the inclusion criteria were met.

ÅS and ML conducted 10 interviews each (between October 2023 and January 2024) and did not interview participants with whom they shared workplace. One person orally consented to participation but dropped out before the interview.

The interviews were conducted individually, face-to face (n 9), digitally (n 9) or by telephone (n 2) at the participants’ workplaces (n 19) or home (n 1), according to the preferences of each participant. All interviews were digitally recorded and transcribed verbatim. Background characteristics of the participants are shown in Table 2. The study was approved by the Swedish Ethical Review Authority (Dnr. 2023-04452-01).

**Table 2.** Characteristics of participants and interviews.

Interview code	Gender	GP/resident GP	Age	Years of work in PHC	Duration of interview	PHCC number	Interview setting	PEth use at PHCC
ML01	M	Resident GP	31	3	38	PCC9	Video	Moderate
ML02	F	Resident GP	40	3	29	PCC8	Physical	Low
ML03	F	Resident GP	36	6	31	PCC1	Video	High
ML04	M	GP	66	39	43	PCC7	Physical	High
ML05	F	Resident GP	33	3	40	PCC7	Physical	High
ML06	F	GP	57	20	30	PCC7	Physical	High
ML07	F	GP	53	21	29	PCC10	Video	Moderate
ML08	F	GP	54	26	34	PCC1	Video	High
ML09	M	GP	58	29	28	PCC9	Video	Moderate
ML10	F	GP	49	18	34	PCC10	Video	Moderate
ÅS01	F	GP	43	11	23	PCC6	Telephone	Low
ÅS02	F	GP	49	9	36	PCC5	Physical	Low
ÅS03	F	Resident GP	39	6	30	PCC6	Physical	Low
ÅS04	M	Resident GP	42	4	37	PCC5	Physical	Low
ÅS05	F	GP	39	11	35	PCC4	Video	High
ÅS06	M	GP	59	24	37	PCC4	Video	High
ÅS07	F	GP	48	21	27	PCC3	Video	Low
ÅS08	M	Resident GP	40	1	45	PCC2	Physical	Low
ÅS09	F	Resident GP	42	4	20	PCC3	Telephone	Low
ÅS10	F	GP	41	12	28	PCC2	Physical	Low

GP: general practitioner; PHC: primary health care; PHCC: primary health care centre.

### Analysis

**Figure 1.**
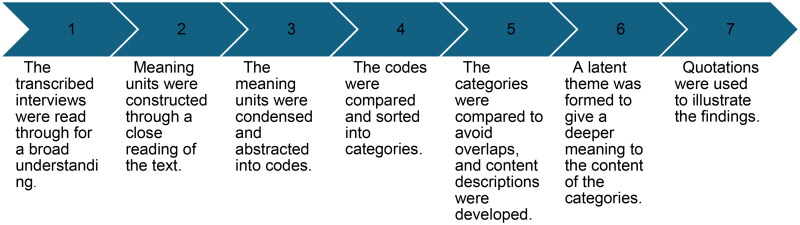
Steps of the analysis.

**Figure 2.**
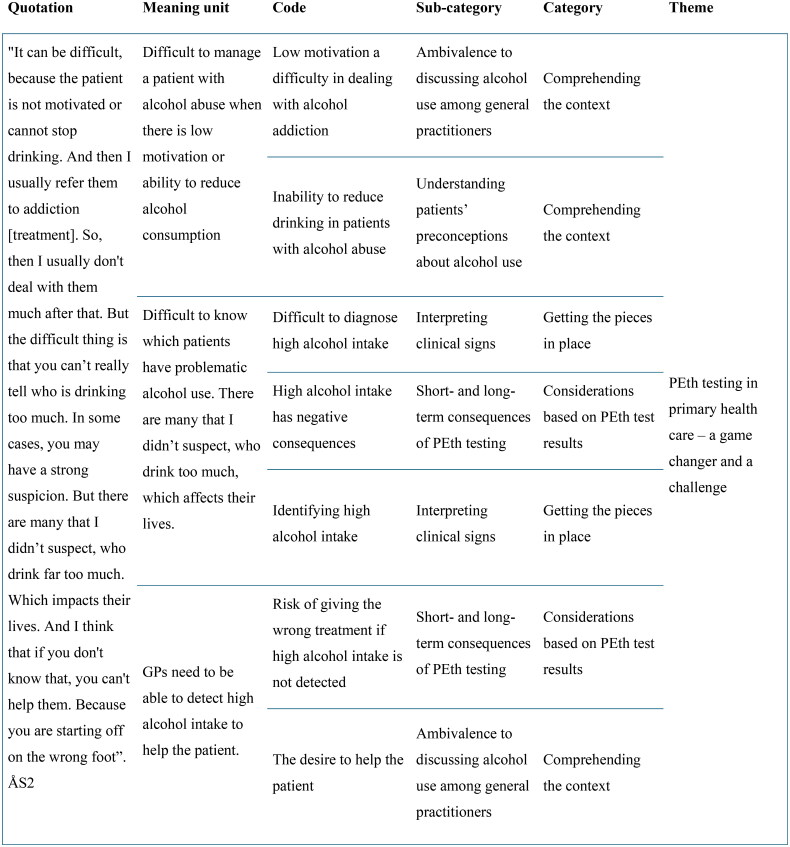
Examples of the steps of the analysis.

The interviews were analysed using latent qualitative content analysis. This method allows for an open-ended analysis with no preconceived categories and enables the categories to emerge from the data as described by Graneheim and Lundman [27]. Figure 1 shows the steps of the analysis. The preliminary categories were mainly coded by ÅS, ML and LK. As part of the reflexivity process, the categories were endorsed by complementing and challenging each other’s readings and preunderstandings [28]. Validity was ensured by testing tentative categories within the entire research group including AJC (female, MD, psychiatrist/addiction specialist, PhD) and AS (female, MD, GP, PhD) in addition to ÅS, LK and ML. The representation of researchers with rich experience in qualitative research and from different disciplines provided an opportunity to validate the findings. Both researchers who conducted the interviews are clinicians with experience in PEth testing. This could have influenced the participants’ answers. However, the interviewers strived to remain neutral throughout the study process and did not interview any of the participants with whom they shared a workplace. Examples of the steps of the analysis are shown in Figure 2.

## Results

**Figure 3.**
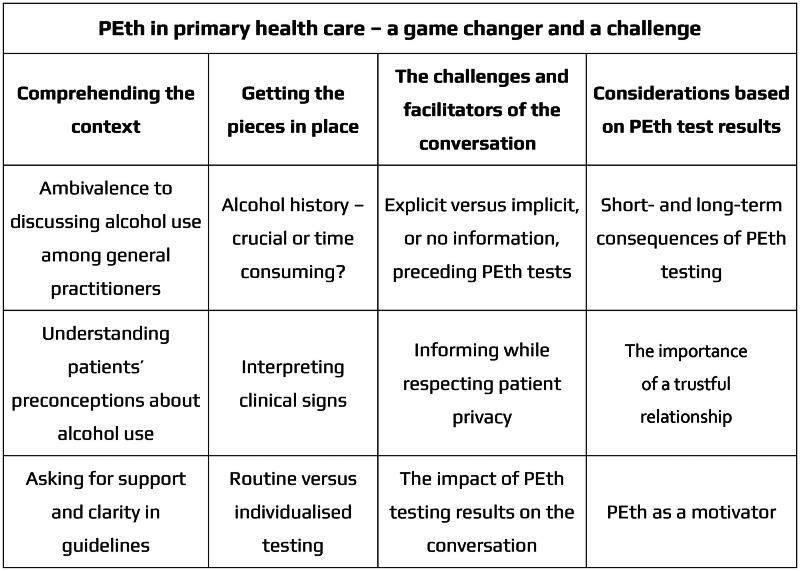
Theme, categories and sub-categories describing the participants’ experiences and perceptions of using PEth tests in primary care.

There was broad variation in terms of age, gender and work experience among the participants (Table 2). During the qualitative content analysis [27], we identified four categories with twelve sub-categories describing the participants’ experiences and perceptions of PEth testing in PHC, namely: *Comprehending the context, Getting the pieces in place, The challenges and facilitators of the conversation* and *Considerations based on the PEth test results*. The latent theme *PEth testing in primary health care—a game change*r *and a challenge* was formed to give a deeper meaning to the content of the categories (Figure 3). Quotations were chosen to illustrate the findings of the study. Capital letters in parentheses at the end of the quotation indicate the interviewer’s initials followed by the participant’s number (Table 2).

### Comprehending the context

This category comprises three sub-categories illustrating the conditions during the doctor’s visit in PHC, where the subject of hazardous alcohol use or AUD was identified and discussed.

#### Ambivalence to discussing alcohol use among general practitioners

Although addressing sensitive topics was considered an essential part of clinical work, a strong ambivalence emerged regarding questions on alcohol. Participants described asking about alcohol habits both as a natural part of history taking and as something sensitive and private. They expressed a fear of shaming or blaming the patients. Some GPs were more prone to emphasise the need to respect and protect patient privacy, while others stated that the patient’s personal privacy was subordinate to the need to address health issues related to hazardous alcohol use or AUD.

#### Understanding patients’ preconceptions about alcohol use

Several participants observed a discrepancy between the alcohol intake reported by the patient and the detected PEth level. The GPs sometimes attributed this to a socially normalised hazardous drinking pattern and low awareness of the health impact of high alcohol intake.

Thanks to this PEth testing, I’ve found so many more than I’ve ever found before, because most people think they drink as much as most people do, and it depends so much on the context they’re in. ML08

Physicians rarely suspected that their patients were being dishonest. In cases where patients provided inaccurate information, the GPs often perceived that they were motivated by a fear of losing access to prescriptions for painkillers or sedatives, or losing their driving licence due to the GP’s obligation to report unfit drivers. The GPs also perceived that some patients underestimate their alcohol intake because of low insight or low motivation to change their drinking patterns.

Due to the patients’ lack of insight or knowledge, the GPs sometimes struggled to find a way to explain the clinical implication of an elevated PEth test result. Participating GPs perceived that their efforts to find explanations were successful when patients showed increased openness to engage in dialogue about alcohol habits.

#### Asking for support and clarity in guidelines

Some participants expressed a wish for clearer guidelines on when and how often PEth tests should be used, how to interpret results and what actions to take when tests show elevated levels. There was also a lack of consensus regarding PEth utilisation, according to the GPs. The participants often relied on their own experience of PEth use and reported that they increased utilisation after they were more familiar with the test. Some also reported difficulties in identifying hazardous alcohol use and diagnosing AUD.

Legal obligations posed another challenge, as GPs in Sweden are obliged to report patients who, due to medical reasons (including moderate to severe AUD) may be unfit to hold a driver’s licence. Some GPs expressed uncertainties regarding to what extent they were required to report unfit drivers. Additionally, the obligation to provide good healthcare was often considered more important than the duty to report.

Another reason for uncertainty were the requirements regarding patient information before a PEth test. Several participants expressed discomfort with informing patients about PEth testing, and some seemed unaware of the guidelines mandating information to patients regarding medical examinations. GPs also found it challenging to balance between the patient’s right to decline a test and the physician’s ability to make an accurate medical assessment, which sometimes required a PEth test.

### Getting the pieces in place

This category comprises three sub-categories describing the physician’s considerations while investigating and treating a patient where the presence of hazardous alcohol use or AUD was considered and how PEth was used in this process.

#### Alcohol history—crucial or time consuming?

Taking alcohol history was in some cases considered important in identifying hazardous use and the PEth test was a useful tool among others. However, some perceived that both the alcohol history taking and the clinical examination could be unreliable. Some even expressed that taking a thorough alcohol history was seldom worth the time. In contrast, PEth testing was regarded as time saving, trustworthy and useful. Therefore, some participants considered that the role of alcohol history taking had changed, from a primarily diagnostic instrument to a tool used to raise the topic of alcohol use.

You can absolutely talk about alcohol, but if you really want to know for certain, then I think testing is better. ÅS02

Furthermore, if a GP prescribes a PEth test after receiving what he or she perceives to be untrustworthy answers, it could raise ethical and relational dilemmas.

It may also be a bit strange when you first ask, ‘How much alcohol do you drink?’ ‘I drink this much. I don’t drink at all.’ Yes, but I will still order an alcohol test for you. ÅS02

#### Interpreting clinical signs

GPs in this study suspected that hazardous alcohol use or AUD was related to several medical conditions, often based on vague clinical signs, for example, when patients did not respond to treatment, recover from mental illness or return to work from sick leave as expected.

It could be that there is another reason for seeking care, and during the interview with the patient, when you take the medical history, you get an idea that something is not right here, it doesn’t add up, and then maybe you get a feeling that there might be something else behind this. And it doesn’t have to be alcohol. It could be something else that one hasn’t been able to pinpoint, but…but… Somewhere there you sort of still want to try to understand if it could be alcohol. ML01

The possibility of AUD was also considered when certain conditions were present, for example hypertension, psychiatric and cardiovascular disorders, substance use disorders including prescribed drug addiction, social problems, and certain laboratory findings.

#### Routine versus individualised testing

Some participants used PEth testing in routine investigations (e.g. psychiatric or cardiovascular disorders), while others used it as part of an individualised assessment. Several obstacles to the routine use of PEth testing were described, such as the relative high cost of testing. However, some estimated the costs of overlooking an AUD as higher compared to the costs of the testing.

Another obstacle was that handling elevated test results was perceived as stressful. Time constraints and limited perceived treatment options for AUD hampered the ability to follow-up after identifying elevated PEth test results. Doubts regarding the usefulness of available interventions also influenced the use of the test, even in cases where AUD was suspected.

### The challenges and facilitators of the conversation

This category comprises three subcategories illustrating the challenges in discussing PEth testing with the patient, as well as the positive impact on the conversation.

#### Explicit versus implicit, or no information, preceding PEth tests

There were varying views and strategies used to handle information on PEth testing. Some participants provided clear information before PEth testing and described using standard phrases to defuse tension around discussing alcohol use or framed PEth testing as a routine procedure to avoid intimidating the patients.

Some found it easier to give information about elevated test results when the patients had consented to the test and therefore informed prior to testing. Others only provided explicit information before testing in cases where the detection of elevated levels would require them to report that the patient was unfit to hold a driving licence or in the case of other negative consequences for the patient. However, other GPs only provided vague or no information about PEth testing, which was seen as part of a routine laboratory work-up. They reported that patients who were not informed about specific tests in a battery showed no negative reactions when informed about PEth test results.

#### Informing while respecting patient privacy

Some of the GPs in this study considered the fact that the patient had booked an appointment for a medical concern as presumed consent to participate in relevant medical investigations. Others stated that PEth testing should be handled cautiously, given that it provides information about lifestyle habits and thus is not only a medical concern, but can also be perceived as an intrusion of the patient’s privacy.

In contrast, some expressed that they know what is best for the patient from a medical perspective. These participants did not consider it necessary to explicitly ask for consent before PEth testing. They expected to be able to better help their patients at an early stage and to treat co-occurring diseases more successfully if the hazardous alcohol use or AUD was identified. Conversely, other GPs reported being reluctant to express their suspicion in order to avoid negative reactions and awkward situations. Many tailored the information they provided to match expectations. In cases where the patient’s autonomy was particularly important to consider, the participants often gave more detailed information before a PEth test.

Depending on how I perceive the patient’s attitude towards coming to a doctor and such. Some are much more like ‘yes, but you take care of the medical part and everything, I don’t care about that, and I will come… I trust you’. And some maybe want to have more control themselves and have stronger privacy there somehow. Yes, they want to know exactly which tests have been done and want to know the results of everything and such. Then maybe it seems more important, I think. ÅS04

#### The impact of PEth test results on the conversation

The PEth test results were a surprise when they did not correlate with the alcohol history. Participating GPs reported striving for an open, honest and non-judgemental conversation and found that those components were more achievable when communicating PEth test results. They perceived PEth as a reliable test that cannot be questioned and more objective than the patient’s report. Factual explanations were considered crucial for patient adherence to follow-up and interventions regarding alcohol habits.

Before, I was uncomfortable talking about this, but with PEth, it’s actually easy. It has probably helped me be more confident, because in some way it is a conclusion or… Yes, I know what I’m talking about all of a sudden. It is much easier to engage in the conversation, and I feel that the patients appreciate that too. ML06

### Considerations based on the PEth test results

This category comprises three subcategories that describe the consequences of identifying elevated PEth levels and patient reactions, and how these affected the GPs’ willingness to use PEth tests.

#### Short- and long-term consequences of PEth testing

Participants perceived that elevated PEth levels generated extra work. In the moment, this could be an obstacle to prescribing the test, despite the awareness that hazardous alcohol use or AUD may negatively influence treatment outcomes over the long term. The GPs and the patients were not always of the same opinion. Hence, interventions seen as necessary from the GPs’ point of view might not be perceived as necessary by the patients. Disagreements could arise when patients request immediate relief, such as prescriptions for anxiety or pain. Furthermore, the participants’ wishes to help their patients were not compatible with the immediate negative effects of potentially losing their driving licence.

Here I am forced to do it [report unfit drivers] when it might not be a … what I see as a real risk. And in addition, it may turn things more upside down, when the patient does not want to seek care when needed, may not even address that particular problem. But it’s clear, if you’re going to try to think broadly, it’s … on the other hand, maybe you can get people to reduce their consumption if their driving licence is at risk. ÅS08

#### The importance of a trustful relationship

Many GPs emphasised the importance of mutual understanding and trust in their relationship with their patients, particularly regarding adherence to suggested interventions. Some participants feared that discussing alcohol habits would upset the patient and therefore harm the relationship. On the other hand, participants reported that they seldom or never experienced any angry reactions related to PEth testing. Some participants reported feelings of failure when the PEth test indicated that patients had not been truthful about their alcohol intake. The GPs also perceived that some patients feared being judged, and that this could prevent them from building a trusting relationship.

That they understand what I mean, that I understand their intention and that we hopefully have some form of trust in that collaboration. Because that is a very important part, and it is very difficult to be someone’s doctor if you don’t have trust or confidence. Both ways somehow. ÅS05

#### PEth testing as a motivator

Informing patients about elevated levels in PEth test results was perceived as a useful tool for motivating patients to decrease their alcohol intake, and a follow-up test was helpful since it provides objective and concrete feedback. Sometimes GPs required a follow-up test before issuing a doctor’s certificate or reporting unfit drivers. These situations often strongly motivated the patient to reduce their alcohol intake. In this sense, legislation regarding driving licences could work as a measure to improve health, although some GPs did not appreciate the obligation to exert control over their patients.

I also think that it can be very motivating for the patient to say ‘now it [PEth] was this and look now, now it [PEth] has gone down like this’, meaning that it becomes measurable in more ways. Partly as a part of realizing that it is overconsumption but also that it is possible to … that you see results when you reduce or halve and so on. ÅS10

### Theme: PEth in primary health care—a game changer and a challenge

The overarching theme was formed to give a deeper meaning to the content of the categories. It runs through the categories and sub-categories, linking them together. *PEth testing in primary health care—a game changer and a challenge* illustrates both the importance of using PEth testing in clinical practice, emerging ethical dilemmas and how these phenomena relate to each other.

The PEth test has provided GPs with a valuable tool for identifying and monitoring patients with hazardous alcohol use. Many participants viewed that PEth should be used more extensively. However, the GPs also struggled to implement this relatively new test and found guidelines to be scarce and effective interventions for hazardous alcohol use and AUD difficult to access.

According to the participating GPs, PEth testing has changed the significance of alcohol history taking, since PEth testing was considered a more reliable, objective and time efficient way to identify high alcohol use. This could be considered a game changer in the doctor-patient conversation about alcohol use in a PHC setting. However, PEth tests had also brought challenges regarding the doctor-patient relationship, according to some of the GPs. While perceiving that there was a medical reason for ordering the PEth test, the GPs sometimes feared that informing about ordering the test would negatively impact the relationship with their patient. A sense of ambivalence often emerged, as the GPs aimed to protect the patient’s privacy while striving to conduct a thorough medical investigation. This manifested in different approaches when the GPs informed patients about ordering the PEth test.

The participants also encountered emotional difficulties when the reported alcohol history did not align with the PEth test results, and the GPs feared that it may harm the relationship with the patient by giving rise to feelings of shame. However, some also stated that elevated PEth levels in a longer perspective often facilitated the conversation and improved the ability to make accurate assessments.

PEth testing could entail undesirable consequences, such as limiting GPs’ ability to prescribe and write doctor’s certificates and require them to report unfit drivers. These difficulties arose primarily when the patients’ insight or motivation to change their drinking habits was insufficient. Meanwhile, when having to take undesirable actions, the GPs considered PEth as a reliable measure for making difficult decisions.

In summary, the PEth test simultaneously improved the prerequisites for the interaction between the GP and the patient in conversations about alcohol use and made it more complex, which is illustrated in the two quotes below.

PEth is an absolutely fantastic test for both us and our patients. And I feel that we have a much better opportunity to help patients with alcohol-related illness after we started using PEth to a greater extent. It’s a bit revolutionary actually. ML10

There is still resistance to taking samples without discussing it with the patient, because it feels like an intrusion in some way, because it doesn’t feel like a purely medical concern. ÅS04

## Discussion

This qualitative study, based on twenty individual interviews with GPs, identifies PEth testing as both an appreciated tool that, according to the participants, has revolutionised the care of patients with hazardous alcohol use and AUD, and as a controversial measure with the potential to give rise to ethical and clinical dilemmas.

Two major ethical dilemmas were identified, which also added new knowledge to the literature. The first dilemma was on how to inform patients before PEth testing. While this dilemma had been identified in a previous study [25], a greater variety of approaches emerged in this study. Interestingly, some of these, such as not informing patients before testing, were at odds with current regulations regarding patient involvement in the provision of healthcare [29].

The second ethical dilemma involved how to interpret legal obligations when treating driving licence holders, which was a novel finding. Other dilemmas concerned the desire to respect patient autonomy and privacy and the need to conduct an appropriate medical investigation and deliver healthcare, especially concerning sedative or pain killer prescriptions and doctor’s certificates when increased PEth levels indicated ongoing AUD. With these obstacles in mind, the participants said that they needed more distinct guidance regarding PEth testing, especially concerning the management and communication of elevated test results.

### Implications for research and practice

Our findings indicate that GPs have a positive view on PEth testing as a complement to alcohol history taking to increase efficiency and accuracy of AUD diagnosis. This is in line with previous research, regarding patients with hypertension in PHC [25], Correct diagnosis was considered an important prerequisite to the delivery of adequate care in several medical conditions, similar to previous findings [25]. Since the identification rate of AUD using only clinical judgement is low [30], implementing diagnostic tools in everyday practice is necessary to improve both early detection of hazardous use, as a preventative measure, and early diagnosis of AUD. Given the results of the current paper and findings from previous research, it would be beneficial if clinical guidelines endorsed structured PEth testing in PHC [31,32]. PEth testing as a possible option in assessment and treatment is briefly mentioned in Swedish guidelines for handling AUD and substance use disorders [33], lifestyle habits [34] and depression and anxiety [6,35]. However, the clinical management of PEth testing is not described. PEth testing as a clinical tool is not mentioned in National Institute for Health and Care Excellence (NICE) [36,37] guidelines or in the World Organization of Family Doctors (WONCA) policy statements [38] although there is an ongoing debate supporting PEth as routine measurement in PHC [32].

Despite two decades of support for implementing the evidence-based concept ‘alcohol screening and brief intervention’ (SBI) in PHC [39,40], it has not been established in routine practice. Some reasons for this are known, including a lack of knowledge and self-efficacy in delivering the interventions [41]. GPs also perceive that the intervention is too extensive, inflexible and difficult to fit into the natural course of consultations [42]. According to the results in this study, a broader introduction of PEth testing in PHC could improve early detection of harmful hazardous alcohol use and AUD, which is the first step in SBI. However, several barriers need to be handled in order to implement the interventions [43].

Similar to findings regarding the clinical implementation of AUDIT [12–14, 5], the GPs identified time constraints, lack of knowledge and the potential lack of treatment options as obstacles to the wider implementation of PEth testing. Another obstacle mentioned is that it is more expensive compared to other routine laboratory tests. However, the GPs considered these costs negligible compared to the additional healthcare costs entailed by undetected AUD. The findings in this study suggest a need for a wider discussion about health economic calculations in order to promote the sustainable use of resources and support GPs in making cost-effective and ethical decisions that yield long-term benefits. Education and local routines regarding PEth testing would support the GPs in their decision-making.

The participants viewed protecting the relationship with their patients as essential, which was described as necessary for achieving patient cooperation in the investigation and treatment of AUD. The GPs strived to avoid intimidating patients and expressed feelings of failure when this occurred. In line with previous research, this indicates that GPs believe that discussing alcohol issues could harm the patient-physician relationship [41]. The participants considered for instance the duty to report driver license holders who presented two elevated PEth test results [44] a significant obstacle in building and maintaining that relationship. These findings imply a need for further training in handling difficult conversations. A peer learning model [45] could be one way to disseminate this knowledge and support hesitant GPs, where colleagues who have found ways to modulate the conversations about alcohol consumption share this knowledge.

Research aiming to explore patients’ experiences and views on PEth testing in primary care is needed and ongoing. This will add further knowledge and is expected to provide information that can facilitate interventions to improve the use of PEth testing in PHC.

### Strengths and limitations

The representation of researchers with rich experience in qualitative research and from different disciplines (PHC, psychiatry and addiction medicine) provided an opportunity to validate the findings. The researchers who conducted the interviews are both clinically active physicians with experience in PEth testing: one GP and one resident in psychiatry. A researcher without previous personal knowledge of the participants conducted the interviews. The fact that the researchers who conducted the interviews are both clinicians with experience in PEth testing could have influenced the participants’ answers. However, the interviewers strived to remain neutral throughout the study process and did not share workplace with any of the participants. Participants were informed when the GP was conducting the interview that her primary role in the interview situation was as a researcher, that she was aware of her preconceptions and aimed to remain neutral throughout the study process. Being interviewed by a fellow GP may also have influenced the participants’ responses and thus be seen as a potential limitation. However, involving researchers with different clinical backgrounds is a strength, since it probably resulted in a broader range of subject matters being highlighted, thus giving a greater depth to the data. All five researchers were involved in the analysis and the results were discussed until an agreement was reached, as part of the validation of the findings, which contributed to trustworthiness. Consolidated criteria for reporting qualitative research (COREQ) [46] were used for reporting the results, which is seen as a strength in relation to trustworthiness [26].

The diverse socioeconomic and geographical conditions, with both urban and rural representation, and the fact that the selection of participants was based on knowledge about variations in PEth test usage at the different workplaces, strengthen the results. In terms of gender, age and work experience from PHC, the participants also varied, which strengthens the credibility of the data [26]. Data collection continued until data redundancy [26] was reached, which was discussed between the two interviewers (ÅS and ML) and the senior researcher with rich experience of qualitative methodology (LK).

Since the present study is a qualitative study, generalisability is not possible. However, we believe that the transferability of our findings is possible to a variety of PHC units where PEth is already in use or intended to be implemented, with special interest in a Scandinavian primary care context. The results of this study might also be of interest for the treatment of patient groups beyond those with hazardous alcohol use or AUD, such as patients with psychiatric or cardiovascular disease.

## Conclusions

In conclusion, this study emphasises that GPs view PEth testing as an important and useful tool, though clinical implementation poses considerable challenges. The two major ethical dilemmas that arise were how to inform a patient appropriately before PEth testing and how to manage the medical and medico-legal obligations that emerge from elevated test results. The participants required further guidance in handling the arising clinical and ethical dilemmas.

## Appendix

### Interview guide

Physicians’ experiences of PEth (Phosphatidylethanol) testing in primary health care

#### Background questions

Age

Sex

Professional title (resident or specialist in general practice)

Year of certification

Number of working years and months in primary care since certification

#### Detection of problem alcohol use

**Broad opening question:** Please, tell me about your experience working with patients where you suspect high alcohol intake based on your work at the healthcare centre.

- How do you ask patients about their alcohol intake?
- Under what conditions or in what situations at the healthcare centre do you think it is important to ask about patients’ alcohol intake?
- How do you proceed if you suspect harmful alcohol use or alcohol dependence?

#### B-phosphatidylethanol (PEth): routines and experiences

##### Broad opening question

Please, tell me about your experiences of PEth testing in patients at the healthcare centre.

- What advantages do you see with PEth testing?
- What challenges do you see with PEth testing?
- What do you do when you detect an elevated PEth value?
- What do you do if a patient does not participate in PEth testing?
- What do you think about the information about PEth testing that is provided to the patient before testing?

#### Comorbidity and rehabilitation

- What does it look like in your practice when it comes to surveying alcohol use in collaboration with other professional categories, such as rehab coordinators and counsellors?
- sssHow does the collaboration with psychiatry, addiction care and other units around patients with alcohol problems work?

#### Conclusion

- Would you like to add anything further regarding your experiences with PEth testing in primary care patients?
